# The effects of multi-nutrient formulas containing a combination of *n*-3 PUFA and B vitamins on cognition in the older adult: a systematic review and meta-analysis

**DOI:** 10.1017/S0007114522001283

**Published:** 2023-02-14

**Authors:** Paul Fairbairn, Simon C. Dyall, Fotini Tsofliou

**Affiliations:** 1 Department of Rehabilitation and Sport Sciences, Bournemouth University, Poole, UK; 2 School of Life and Health Sciences, University of Roehampton, London, UK

**Keywords:** *n*-3 PUFA, B vitamins, Cognition, Memory, Older adults

## Abstract

There is now evidence to suggest that there may be an interaction between B vitamins and *n*-3 PUFA, with suggestions that increasing intake of both nutrients simultaneously may benefit cognition in older adults. The aim of this systematic review was to investigate whether supplementation with a combination of *n*-3 PUFA and B vitamins can prevent cognitive decline in older adults. Randomised controlled trials conducted in older adults that measured cognitive function were retrieved. The included trials provided a combination of *n*-3 PUFA and B vitamins alone, or in combination with other nutrients. Trials that provided *n*-3 PUFA alone and also measured B vitamin status or provided B vitamin supplementation alone and measured *n*-3 PUFA status were also included. The databases searched were The Cochrane Library, EMBASE, CINAHL, Scopus and MEDLINE. A total of 14 papers were included in the analysis (*n* 4913; age: 60–70 years; follow-up 24 weeks to 4 years). The meta-analysis results found a significant benefit of nutrient formulas, which included both *n*-3 PUFA and B vitamins alongside other nutrients, *v*. placebo on global cognition assessed using composite scores from a neuropsychological test battery (G = 0·23, *P* = 0·002), global cognition using single measures of cognition (G = 0·28, *P* = 0·004) and episodic memory (G = 0·32, *P* = 0·001). The results indicate that providing a combination of *n*-3 PUFA and B vitamins as part of a multi-nutrient formula benefits cognition in older adults *v*. a placebo, and the potential for an interaction between these key nutrients should be considered in future experimental work.

In Europe, the proportion of adults aged 65 years and over is expected to rise from 16·1 to 22 % by 2031^(1)^. It has been estimated that in the year 2015, 688, 300 people in England had dementia with 6·7 % of older adults having the condition^(2)^. Due to the insidious onset, cognitive impairment often goes unnoticed for several years, with clinical diagnosis being made late into the disease progression, thus it is likely that a much higher number of older adults are currently living with some form of cognitive impairment^(3)^. Cognition is critical for functional independence as people age, including whether someone can live independently, manage finances, take medications correctly and drive safely. In addition, intact cognition is vital for humans to communicate effectively, including processing and integrating sensory information and responding appropriately to others^(4)^.

Within the body of observational literature, some key nutrients have been identified as being protective of brain health in ageing populations, with *n*-3 PUFA and B vitamins showing particular promise^(5,6)^. These observations are supported by mechanistic work that has demonstrated that *n*-3 PUFA can reduce markers of inflammation and influence membrane properties, which in turn affects cell signalling, increases neurogenesis and promotes neuronal survival^(7–9)^, with vitamins B_12_, B_6_ and folic acid playing essential roles in the metabolism of homocysteine preventing hyperhomocysteinaemia, which has been strongly linked to cognitive decline in older adults^(10,11)^.

Despite the consistent findings within the epidemiological literature, randomised control trials focusing on supplementation with *n*-3 PUFA or B vitamins in isolation have produced inconsistent and inconclusive results^(12–18)^. Whilst there is a great degree of heterogeneity in the way randomised control trials have been designed within this area including the dosage of supplementation, length of follow-up, population characteristics and chosen outcomes one characteristic that is a common choice is to supplement with *n*-3 PUFA or B vitamins alone. Whilst this approach allows us to draw causal inferences for specific nutrients, a major limiting factor of this approach is that single nutrients often have very small effect sizes that could be influenced by bias and could lack clinical significance^(19)^. Indeed, whilst the observational literature has found positive relationships between single nutrients and cognitive outcomes, it must be acknowledged that the intake of these nutrients has predominately come from whole food sources which are of course sources of many other essential nutrients which may have also contributed towards such associations.

It is now clear that there are complex interactions between several nutrients that make up a balanced diet and that it is important to explore potential synergistic or additive effects to elicit how whole dietary interventions can impact a specific outcome. For instance, some evidence suggests that there may be an interaction between B vitamins and *n*-3 PUFA, and optimising intake of both nutrients may be key to eliciting beneficial effects on cognition^(20,21)^.

A lack of consideration for this potential interaction between *n*-3 PUFA and B vitamins could contribute somewhat to the variance in results from single nutrient supplementation trials. It is therefore important to examine the current available literature that has investigated the combined effects of *n*-3 PUFA and B vitamins. As such, the aim of this systematic review was to first investigate whether supplementation with a combination of *n*-3 PUFA and B vitamins alone or as part of a multi-nutrient formula can prevent cognitive decline in older adults. Second, the review sought to determine whether the effects of a single nutrient intervention with either *n*-3 PUFA or B vitamins could be modified by the status of the other nutrient.

## Methods

This systematic review was registered and made available to the public through The International Prospective Registration of Systematic Reviews (https://www.crd.york.ac.UK/PROSPERO)(CRD42020210361).

The research question was developed using the population, intervention, control and outcome tool. The population of focus was older adults defined as being aged 60 years and older, the intervention was nutrient formulas containing both *n*-3 PUFA and B vitamins either alone or in combination with other nutrients, the control was placebo supplementation and the outcomes were global cognition, episodic memory and executive function as measured by neuropsychological testing.

### Search strategy

The following databases were searched in December 2020 for articles published between January 2010 and December 2020: the Cochrane Library, EMBASE, CINAHL, Scopus and MEDLINE. The search strategy used terms related to the combination of *n*-3 PUFA (*n*-3 polyunsaturated fatty acid, *n*-3 PUFA, *n*-3 PUFA, eicosapentaenoic acid, EPA, docosahexaenoic acid, DHA, fish oil) and B vitamins (B vitamin, vitamin B, B_12_, folic acid, folate, homocysteine, cobalamin, multivitamin and multi-nutrient). The key words were then combined by the EBSCO host operator AND/OR. Supplementary literature searches included examining the reference lists of all relevant studies, pertinent review articles and meta-analyses.

### Study selection criteria

Articles were included if they met the following criteria: (1) the study type was a randomised controlled trial and was available in the English language; (2) the mean age of the participants in the study was 60 years or greater; (3) the study intervention provided a combination of *n*-3 PUFA and B vitamins alone or in combination with other nutrients; (4) tested for interactions between *n*-3 PUFA and B vitamins by providing *n*-3 PUFA alone but also including an objective measure of B vitamin status including measuring B vitamins directly or through assessment of homocysteine, provided B vitamin supplementation alone but also measured an objective measurement of *n*-3 PUFA status and (4) assessed cognitive function through a change in a composite score on neuropsychological testing or change in single cognitive test score. Randomised control studies are able to provide a controlled and consistent dosage of nutrients to participants and allows for causal inferences to be made thus the decision was made to limit the included studies to this more robust design. The articles were excluded if they met one of the following criteria: (1) duplicated publications; (2) non-randomised control trials including letters, case reports, position statements, conference proceedings, prevalence surveys, reviews, *in vitro* studies and studies in animals and (3) studies that did not report specific dosages for individual nutrients provided in supplements. The initial screening was performed by the lead researcher (PF) and included a review of all titles and/or abstracts compared with eligibility criteria, with consensus sought from a second researcher (FT) where there was ambiguity. Full-text publications of any studies not eliminated within the initial screening were retrieved for complete review.

### Data extraction

Data extraction and coding stages of the review were completed by the first reviewer (PF) using structured data extraction forms. The following information was extracted from the manuscripts: first author, year of publication, location, number of participants, duration of intervention, age, the intervention (dose and formulation of dietary supplements), method used to assess cognitive testing, the cognitive domains that were tested and any additional relevant biomarkers that were measured (*n*-3 PUFA, B vitamin or homocysteine levels). A proportion of the extracted data (30 %) was checked for accuracy by the second reviewer (FT).

### Risk of bias in individual studies

Risk of bias was assessed using the Revised Cochrane risk-of-bias tool for randomised trials (RoB 2) which comprises the respective RoB2 domains: (1) risk of bias arising from the randomisation process; (2) risk of bias due to deviations from the intended interventions (effect of assignment to intervention); (3) risk of bias due to missing outcome data; (4) risk of bias in the measurement of the outcome and (5) risk of bias in the selection of the reported result^(22)^. These domains were used to inform the overall risk-of-bias judgement for the selected studies. PF independently assessed the risk of bias for all included studies. FT performed an independent check on the risk-of-bias scores to ensure the accuracy of scoring. Disagreements were resolved by discussion, and a third opinion was sought from an independent researcher where there was a discrepancy.

### Data synthesis

Effect sizes were based on group mean differences (post-study minus pre-study test scores) and corresponding SD between the combined *n*-3 PUFA and B vitamin intervention group and the control (placebo) group. When SD were not reported, methods described in the Cochrane Handbook for Systematic Reviews of Interventions were relied upon to calculate or estimate SD from other statistics provided in the published paper. For each effect size, the Hedges G statistic was calculated. This approach permits combining multiple methods of cognitive testing for which different scales were used to determine scores. This effect size is a variation on Cohen’s d, which corrects for biases due to small sample sizes^(23)^. The scales of cognitive tests were made to have a consistent direction of effect across all included studies, with positive estimates favouring intervention groups and negative estimates favouring placebo groups. Random-effects meta-analysis models were used to generate between group effect sizes.

*A priori* analyses were defined for between groups. Macro-level models included data on all subjects, regardless of baseline cognitive status or age, at all dose levels using the longest duration of exposure for each cognitive domain. The primary analysis included studies that had used a composite score for cognition using a neuropsychological test battery to assess overall cognitive status. It has become evident that commonly used tests to assess global cognition such as the mini-mental state examination and Alzheimer’s Disease Assessment Scale-cognitive subscale are not adequate to detect changes particularly in healthy older adults or those with mild cognitive impairment^(24)^. As such, the use of a neuropsychological test batteries is increasingly seen as a promising method to detect changes in cognition at an early stage of cognitive decline^(24)^. Secondary analyses were performed on single tests of global cognition as well as episodic memory and executive function. Episodic memory and executive function are commonly assessed cognitive domains within the literature and have been demonstrated to be sensitive indicators of pathological cognitive decline^(25,26)^. Where multiple single tests of global cognition were used, the primary outcome was selected for use in the meta-analysis. The weight of each study in the meta-analysis was based on the inverse of the variance, a measure that accounts for the sample size within each group. The statistical heterogeneity among studies was determined according to the *χ* ^2^ test and I^2^ statistic, with *P* > 0·1 and I^2^ < 50 % was considered as low heterogeneity, and otherwise it indicates high heterogeneity when the I^2^ value was higher than 50 %.

A sub-group analysis was performed based on the participant’s cognitive health status at baseline. Previous studies have indicated that patients with milder cognitive symptoms may more likely respond to supplementation with either *n*-3 PUFA or B vitamins than those with diagnosed Alzheimer’s disease^(14,27)^. For this sub-group analysis, participants could be classified as healthy without any cognitive impairment, prodromal indicated by mild cognitive impairment, which was inclusive of self-reported or objectively measured cognitive impairment and finally diagnosed Alzheimer’s disease.

The exploratory post hoc analyses of previous studies providing single nutrient interventions aimed to detect interactions between supplementation and background diet thus were conducted and analysed in a different manner to the trials that provided combined *n*-3 PUFA and B vitamin interventions. As such, it would not be appropriate to include these studies within the meta-analysis alongside the interventions trials that have provided a combination of nutrients. These studies have been descriptively summarised.

## Results

**Fig. 1.**
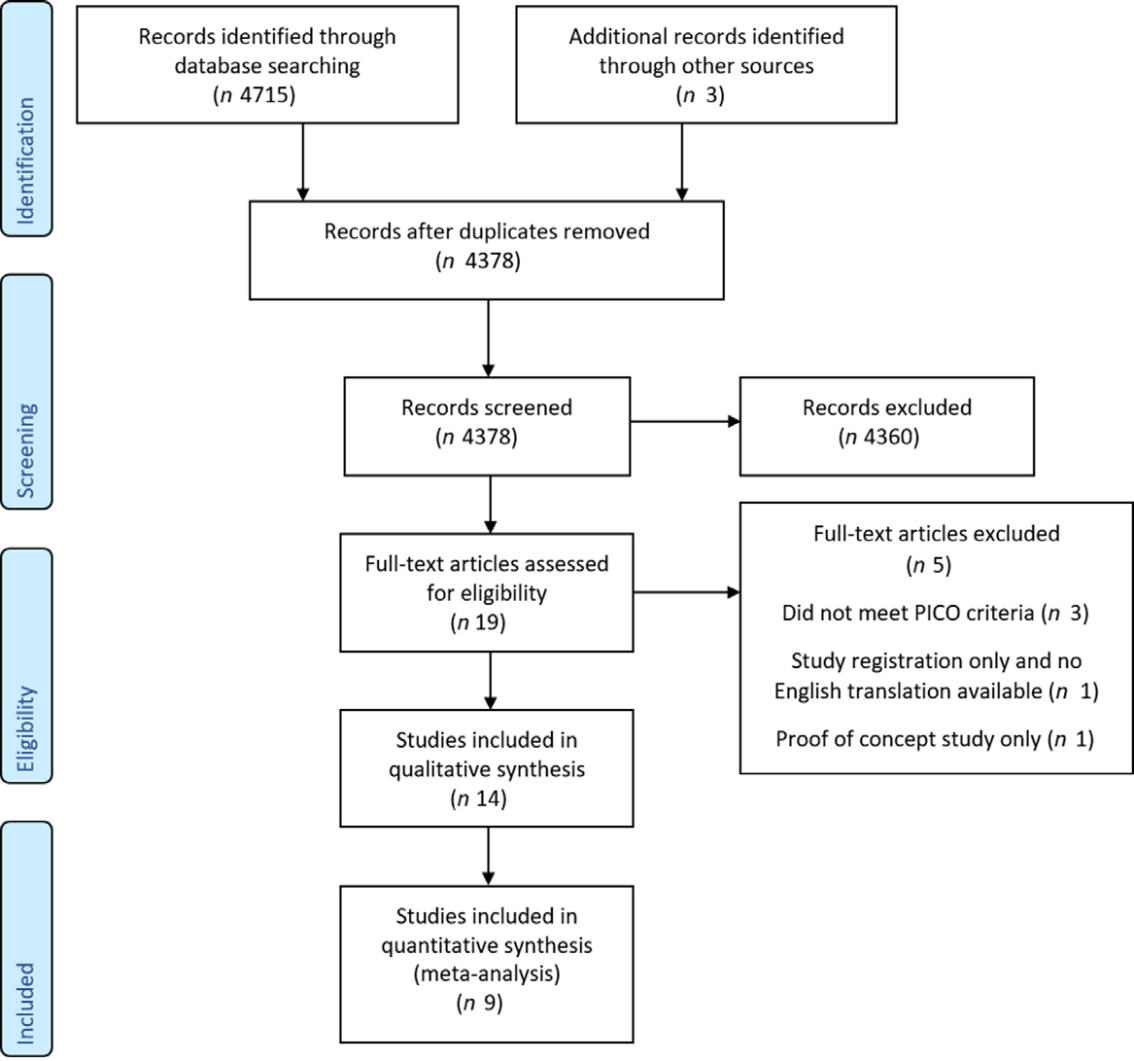
PRISMA flow chart showing the literature screening process and study selection.

The databases identified 4715 results with three additional sources coming from forward citation searching (Fig. 1). After the removal of duplicates, 4378 articles remained. The titles and abstracts of these articles were screened, 4, 359 articles were excluded, with the 19 remaining articles undergoing a full-text screening. A further five articles were excluded at this stage. One study had a mean age of below 60 years^(28)^, two studies had no measurement of cognitive testing through neuropsychological testing^(20,29)^, one study was not available in the English language, this study was only logged on a trial registry and a full text was not made available for translation and one study was a proof of concept study thus was not designed for hypothesis testing^(30)^.

### Characteristics of included studies

A full summary of the characteristics of each included study can be found in Table 1. All of the included studies were randomised placebo-controlled studies with follow-up lengths varying between 24 weeks and 4 years.

**Table 1.** Characteristics extracted from fourteen included studies

Author	Age and number of participants	Participants in study	Nutrient interventions/post hoc nutrient measurements	Intervention duration	Cognitive domains tested
Primary Studies					
Fairbairn *et al.*, 2020^(38)^	Mean age 67 ± 7 y *n* 25	Healthy older adults	Active: 1 g DHA, 160 mg EPA, 240 mg *Ginkgo biloba*, 60 mg phosphatidylserine, 20 mg D-α tocopherol, 1 mg folic acid and 20 μg vitamin B_12_ Placebo: fatty acid blend typical of UK. Diet	24 weeks	Episodic memory Executive function Interference control Working memory
Strike *et al.*, 2016^(31)^	Mean age 66 ± 5 y *n* 29	Healthy older adults	Active: 1 g DHA, 160 mg EPA, 240 mg *Ginkgo biloba*, 60 mg phosphatidylserine, 20 mg D-α tocopherol, 1 mg folic acid and 20 μg vitamin B_12_ Placebo: fatty acid blend typical of UK. Diet	24 weeks	Episodic memory Executive function Processing speed
Jackson *et al.*, 2016^(32)^	Mean age 60 ± 5 y *n* 100	Older adults with subjective memory deficits in the absence of cognitive impairment (MMSE scores ≥ 26)	Active: 1 g DHA, 160 mg EPA, 240 mg *Ginkgo biloba*, 60 mg phosphatidylserine, 20 mg D-α tocopherol, 1 mg folic acid and 20 μg vitamin B_12_ Active: 896 mg DHA, 128 mg EPA Placebo: placebo: fatty acid blend typical of UK. diet	6 months	Working memory Processing speed Mental fatigue
Baleztena *et al.*, 2018^(33)^	*n* 99 Mean age 87 ± 6 y	Healthy older adults	Active: 750 mg DHA, 120 mg EPA, 15 mg vitamin E, 45 mg phosphatidylserine, 285 mg tryptophan, 15 μg vitamin B_12_, 750 μg folate and 180 mg *Ginkgo Biloba* Placebo: Gelatin capsule		Global cognition Semantic memory Executive function
Soininen *et al.*, 2021^(39)^	*n* 162 Mean age 71 ± 7 y	Prodromal Alzheimer’s disease, defined according to the IWG-1 criteria	Active: Fortasyn blend: 300 mg EPA, 1200 mg DHA, 106 mg phospholipids, 400 mg choline, 625 mg uridine monophosphate, 40 mg vitamin E, 80 mg vitamin C, 60 µg selenium, 3 µg vitamin B_12_, 1 mg vitamin B_6_, and 400 µg folic acid. Placebo: isoenergetic drink	36 months	Global cognition Episodic memory Executive function
Soininen *et al.*, 2017^(34)^	Mean age 71 ± 7 y *n* 311	Prodromal Alzheimer’s disease, defined according to the IWG-1 criteria	Active: Fortasyn blend: 300 mg EPA, 1200 mg DHA, 106 mg phospholipids, 400 mg choline, 625 mg uridine monophosphate, 40 mg vitamin E, 80 mg vitamin C, 60 µg selenium, 3 µg vitamin B_12_, 1 mg vitamin B_6_, and 400 µg folic acid. Placebo: isoenergetic drink	24 months	Global cognition Episodic memory Executive function
Shah *et al.*, 2013^(35)^	*n* 527 Mean age: 77 ± 8 y	Older adults with mild to moderate Alzheimer’s disease receiving Alzheimer’s medications (Acetylcholinesterase inhibitor, Memantine or combination)	Active: Fortasyn blend: 300 mg EPA, 1200 mg DHA, 106 mg phospholipids, 400 mg choline, 625 mg uridine monophosphate, 40 mg vitamin E, 80 mg vitamin C, 60 µg selenium, 3 µg vitamin B_12_, 1 mg vitamin B_6_ and 400 µg folic acid. Placebo: isoenergetic drink	24 weeks	Global cognition Attention and concentration Executive function Processing speed Semantic memory
Scheltens *et al.*, 2012^(37)^	*n* 259 Mean age 74 ± 7 y	Mild Alzheimer’s patients, MMSE scores 20–26, without medications	Active: Fortasyn blend: 300 mg EPA, 1200 mg DHA, 106 mg phospholipids, 400 mg choline, 625 mg uridine monophosphate, 40 mg vitamin E, 80 mg vitamin C, 60 µg selenium, 3 µg vitamin B_12_, 1 mg vitamin B_6_ and 400 µg folic acid. Placebo: isoenergetic drink	24 weeks	Global cognition Memory Executive function
Scheltens *et al.*, 2010^(36)^	*n* 225 Mean age 74 ± 8 y	Mild Alzheimer’s patients, MMSE scores 20–26, without medications	Active: Fortasyn blend: 300 mg EPA, 1200 mg DHA, 106 mg phospholipids, 400 mg choline, 625 mg uridine monophosphate, 40 mg vitamin E, 80 mg vitamin C, 60 µg selenium, 3 µg vitamin B_12_, 1 mg vitamin B_6_ and 400 µg folic acid. Placebo: isoenergetic drink	12 weeks	Global cognition Episodic memory
Li *et al.*, 2020^(41)^	*n* 240 Mean age 70 ± 7 y	Older adults with MCI according to DSM-5	Active: 800 μg folic acid and 800 mg DHA Active: 800 μg folic acid Active: 800 mg DHA Placebo: soybean oil	6 months	Global cognition Executive function Working memory Semantic memory Attention and concentration
Andreeva *et al.*, 2011^(40)^	*n* 1748 Mean age 61 ± 9 y	Cognitively healthy adults who have had a cardiovascular event (myocardial infarction, unstable angina or ischaemic stroke	Active: 560 µg folate, 3 mg vitamin B_6_, 20 µg vitamin B_12_ and 400 mg EPA and 200 mg DHA Active: 560 µg folate, 3 mg vitamin B_6_ and 20 µg vitamin B_12_ Active: 400 mg EPA and 200 mg DHA Placebo: gelatin capsule	4 years	Global cognition
Post hoc analyses					
Oulhaj *et al.*, 2016^(21)^	Mean age 77 ± 5 y *n* 266	Older adults with MCI	Active 800 µg folic acid, 0·5 mg vitamin B_12_ and 20 mg vitamin B_6_ Placebo: placebo tablet Post hoc measurement: plasma *n*-3 PUFA	2 years	Global cognition Episodic memory
Jernerén *et al.*, 2019^(42)^	Mean age 77 ± 4 y *n* 171	Older adults with Alzheimer’s disease treated with acetylcholinesterase inhibitors	1·7 g DHA and 600 mg EPA Placebo: isoenergetic placebo oil (1 g of corn oil, including 0·6 g of linoleic acid) Post hoc measurement: Plasma homocysteine	6 months	Global cognition
Van Soest *et al.*, 2021^(43)^	Mean age 60·2 ± 5·6 *n* 791	Cognitively healthy adults with elevated plasma homocysteine (≥ 13 μmol/l)	800 μg folic acid Placebo: placebo tablet Post hoc measurement: plasma cholesteryl ester *n*-3 PUFA	3 years	Global cognition Episodic memory Processing speed Executive function

DSM, The Diagnostic and Statistical Manual of Mental Disorders, Fifth Edition; IWG-1, International Working Group-1; MCI, mild cognitive impairment; MMSE, mini mental state examination; y, year.

### Nutrient interventions and formulas

Eleven studies were randomised controlled trial (RCT) analysing the effects of a combination of *n*-3 PUFA and B vitamins. Of these eleven studies, nine provided multi-nutrient supplement formulas that included additional active ingredients beyond just *n*-3 PUFA and B vitamins^(31–39)^. Two studies provided only a combination of B vitamins and *n*-3 PUFA with no additional active ingredients^(40,41)^. Three of the included studies included additional intervention arms that provided single-nutrient interventions of either *n*-3 PUFA or B vitamins that allowed for comparison between the single-nutrient and multi-nutrient formulas^(32,40,41)^. Three included papers were post hoc analyses of RCT, one was a study that provided *n*-3 PUFA supplements and proceeded to measure homocysteine^(42)^, with two providing B vitamin supplements and proceeding to measure *n*-3 PUFA status^(21,43)^. All of the post hoc studies analysed the interaction between *n*-3 PUFA and B vitamins on cognitive outcomes.

### Participant health status at baseline

Participant’s health status at baseline varied between the studies particularly in regard to cognitive health status. Three of the studies included participants who were cognitively healthy at baseline^(31,33,38)^. One study included participants with a subjective memory complaint^(32)^. Four studies included participants who were at the prodromal stage of Alzheimer’s Disease or had mild cognitive impairment (MCI)^(21,34,39,41)^, with four studies having participants with diagnosed Alzheimer’s Disease^(35–37,42)^. Of the studies that included participants with Alzheimer’s Disease two were in drug naive patients^(36,37)^ with the other two being in patients taking medications with taking either acetylcholinesterase inhibitors, an N-methyl-D-aspartate receptor antagonist or a combination of the two^(35,42)^. The final study included participants who were cognitively healthy at baseline but had suffered a cardiovascular event (myocardial infarction, unstable angina or ischaemic stroke)^(40)^.

### Measurement of cognitive function

A variety of methods were used to quantify cognitive function across all studies. Two studies used measures of global cognition only^(40,42)^, with eleven studies electing to use some domain-specific testing^(31–39,41)^. Four studies used a composite score of multiple cognitive testing methods to quantify overall global cognition^(34,35,37,39)^. The majority of the studies (*n* 11) included a measurement of global cognition. With regard to domain-specific cognitive testing, there was a notable variance in the selected domains that were selected for assessment. The domains that were tested were executive function (*n* 9), episodic memory (*n* 7), processing speed (*n* 3), semantic memory (*n* 3), working memory (*n* 3), attention and concentration (*n* 2), interference control (*n* 1) and mental fatigue (*n* 1).

### Quality assessment

**Fig. 2.**
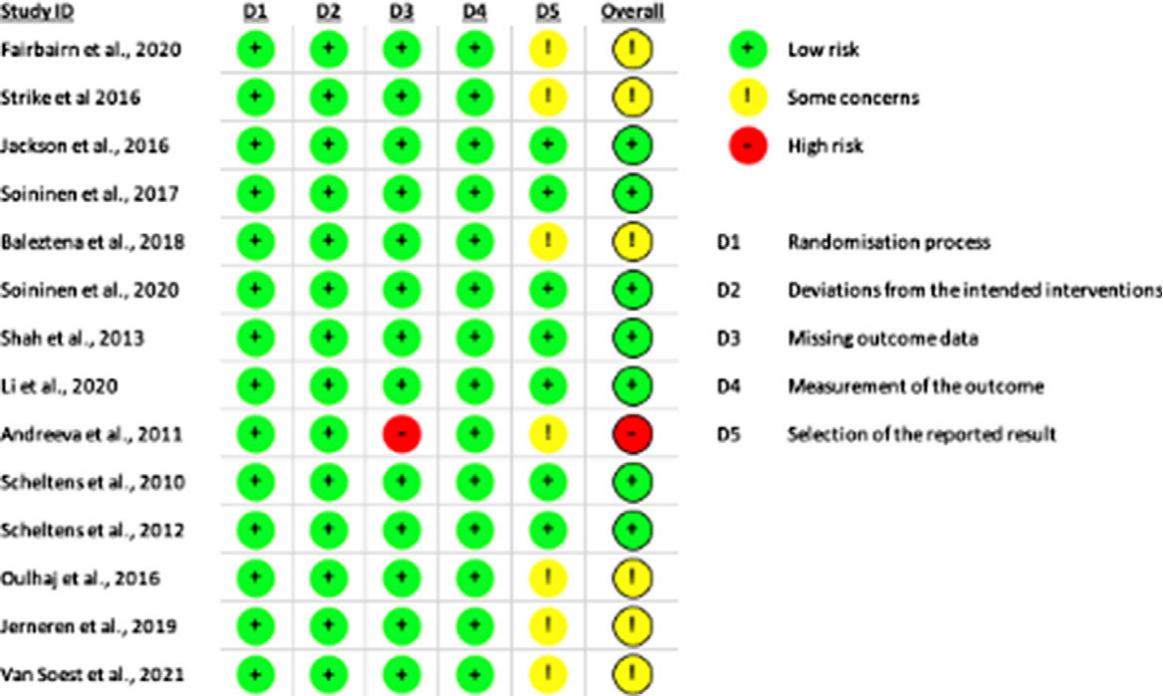
Individual study results from the Cochrane risk-of-bias tool for randomised trials version 2 (RoB 2). In this colour-coded ranking, green colour represents low risk of bias, yellow some concerns and red high risk of bias.

A summary of the risk-of-bias assessment from the RoB 2 can be found in Fig. 2.

### Findings of the included studies

Within the studies that used multi-nutrient formulas that included ingredients beyond *n*-3 PUFA and B vitamins, three different nutrient formulas were subject to testing. Five of these publications were derived from testing of Fortasyn Connect nutrient blend. The early human trials of the Fortasyn Connect blend were performed in drug naive mild Alzheimer’s Disease patients (formal diagnosis of probable Alzheimer’s Disease and mini mental state examination score > 20) and yielded positive results^(36,37)^. Both of these studies showed positive effects of the nutrient intervention *v*. placebo on memory; however, there were no significant effects on other cognitive domains and tests that included executive function, Alzheimer’s Disease Assessment Scale-cognitive subscale and the twelve-item Neuropsychiatric Inventory. Following on from these initial human trials in drug naive Alzheimer’s Disease patients a study in 527 participants who were taking Alzheimer’s Disease medications found that 24 weeks of the Fortasyn Connect blend had no effect on Alzheimer’s Disease Assessment Scale-cognitive subscale, attention and concentration, executive function, processing speed or semantic memory^(35)^. Within 311 participants at the prodromal stage of Alzheimer’s Disease, following a 24-month intervention with the Fortasyn Connect blend, there were no significant effects on the composite score for the neuropsychological test battery as well as composite scores for episodic memory and executive function in both intention to treat and per-protocol analysis; however, a positive effect was observed on the clinical dementia rating score with a difference in the rate of decline of 0·6 *v*. the placebo^(34)^. This study was subsequently extended by an additional 12 months which included 162 of the original participants. After 36 months of supplementation, there was a significant improvement in the composite score for the neuropsychological test battery, composite episodic memory and clinical dementia rating all with small effect sizes based on the Cohens D statistic^(39)^.

Outside of the work conducted on the Fortasyn Connect blend, six other RCT were identified that provided participants with a blend of *n*-3 PUFA and B vitamins. The SU.FOL.OM3 trial tested the effects of B vitamins and *n*-3 PUFA supplementation alone and in combination in a factorial design^(44)^. The study was initially designed to detect changes on cardiovascular events; however, ancillary findings have been published on cognition where sub-group analysis was performed to focus on participants aged 65–80 years. The authors found that in the participants aged 65–80 years, B vitamin supplementation alone was associated with a lower global cognition with no other effects of supplementation in any other group. In a similar factorial design, which provided DHA and folic acid alone and in a combined formula in a sample of 240 older adults with MCI, the combined intervention led to a significant improvement in global cognition *v*. placebo with each intervention alone providing no significant benefit to this domain. Improvements in executive function and working memory were also observed for the combined DHA and folic acid intervention^(41)^.

The effects of a high DHA multi-nutrient supplement containing a daily dosage of 1000 mg DHA, 160 mg EPA, 20 µg vitamin B_12_, 1 mg folic acid, 124 mg PS, 20 mg vitamin E and 240 mg *Ginkgo Biloba* standardised leaf extract in older adults have been examined in three RCT. Strike *et al.* found that 24 weeks of dietary supplementation resulted in improved episodic memory and processing speed *v*. placebo in a sample of twenty-seven healthy older adults. A subsequent 24-week RCT from this lab found improvements in episodic memory and executive function within a sample of twenty-five healthy older adults^(38)^. The effects of this nutrient formula were also examined in a sample of 100 older adults with subjective memory complaints for 24 weeks. As well as having the multi-nutrient supplement and placebo interventions, the researchers also added a third group that took a similar dose of fish oil alone (896 mg DHA and 126 mg EPA) to assess whether the addition of the supporting nutrients influenced the results. The primary outcome of this study was cerebral blood flow in the prefrontal cortex with cognitive tests that are specific to this region being included as secondary outcomes. Following the intervention period, there was no effect of the multi-nutrient formula or *n*-3 PUFA alone on cerebral blood flow or cognitive outcomes^(32)^. The final identified RCT examined the effects of a nutrient blend containing a daily dosage of 750 mg DHA, 120 mg EPA, 15 mg vitamin E, 45 mg phosphatidylserine, 285 mg tryptophan, 15 μg vitamin B_12_, 750 μg folate and 180 mg *Ginkgo Biloba*. The study included 99 older adults who were either cognitively healthy or were showing signs of MCI, assessed via the Global Deterioration Scale. No significant effects of supplementation were detected across the tested cognitive domains which included global cognition, executive function and semantic memory.

Three published articles from post hoc analyses of previous primary data sets were identified. The first was from The Vitamins in Cognitive Impairment (VITACOG) trial by Smith and co-workers^(14)^, a study on B vitamin supplementation, which reanalysed the data on cognitive function based on participants plasma *n*-3 PUFA levels. Participants from the study by Smith and co-workers^(14)^, which provided B vitamin supplementation (daily dose 0·8 mg folic acid, 20 mg B_6_ and 0·5 mg B_12_) to 187 older adults with MCI, were divided into tertiles based on their plasma *n*-3 PUFA levels at baseline. The authors found that total EPA + DHA above 590 μmol/l in conjunction with B vitamin supplementation improved episodic memory, global cognition and clinical dementia rating score, whereas participants with *n*-3 PUFA levels of less than 390 μmol/l showed no benefits of B vitamin supplementation^(21)^. When results from these analyses were stratified by EPA and DHA separately, DHA alone but not EPA led to significant effects on clinical dementia rating and episodic memory. In a similarly designed post hoc analysis of the Folic Acid and Carotid Intimamedia Thickness (FACIT) trial, where 791 women with elevated plasma homocysteine (≥ 13 μmol/l) were provided with 800 μg folic acid for a period of three years, participants were stratified by *n*-3 PUFA content in cholesterol esters. Positive interactions were detected between folic acid supplementation and *n*-3 PUFA levels for global cognition and processing speed; however, these interactions were only observed within the lower tertile of *n*-3 PUFA, indicating that folic acid supplementation was only effective whilst accompanied by lower levels of circulating *n*-3 PUFA^(43)^. The final post hoc analysis reanalysed results from a trial on *n*-3 PUFA supplementation, the OmegAD study by Freund-Levi *et al.*, according to baseline plasma homocysteine levels. The OmegAD trial provided 171 older adults with Alzheimer’s Disease with *n*-3 PUFA supplements containing 1720 mg DHA and 60 mg EPA for six months. Overall, no effects of supplementation were observed on cognitive outcomes. In the post hoc analysis, participant’s baseline plasma homocysteine was measured to determine whether this modified the response to *n*-3 PUFA supplementation. The results from the analysis showed that those in the active *n*-3 PUFA supplementation group who also had homocysteine levels < 11·7 mol/l had a 7·1 % improvement in their mini mental state examination scores *v*. those in the same homocysteine tertile assigned to the placebo supplement. A similar result was found for clinical dementia rating sum of boxes score with improvements of 22·3 %^(42)^.

### Funding sources

Ten of the included studies received funding or support from the manufacturers of the supplements that were being tested^(31,32,34–39,41,42)^, three studies did not receive funding from supplement manufacturers^(21,33,43)^ and one study did not clearly report the precise sources of funding^(40)^.

### Meta-Analysis results

**Fig. 3.**
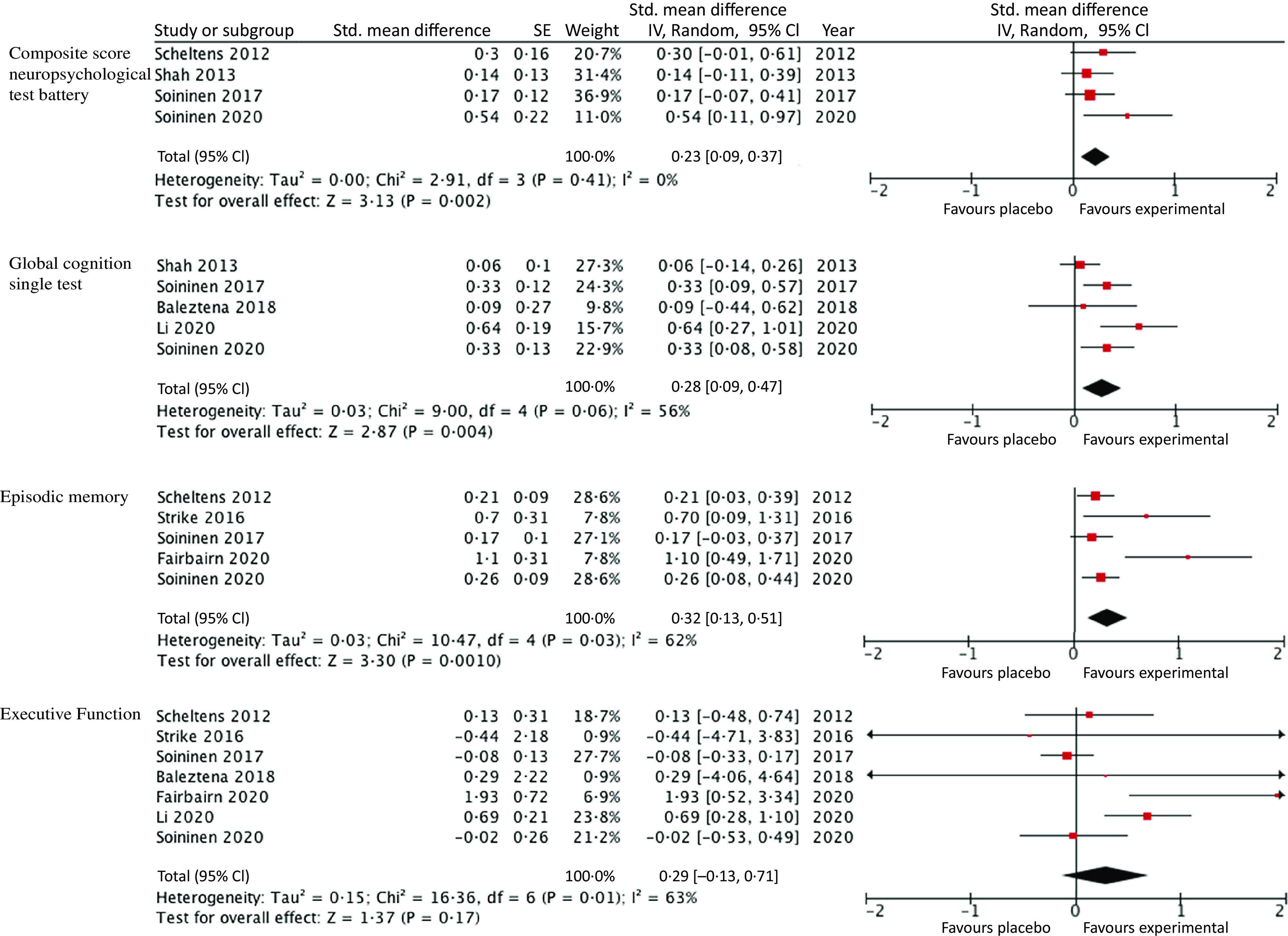
Meta-analysis and forest plots for the effects of multi-nutrient interventions containing both *n*-3 PUFA and B vitamins on composite scores from neuropsychological test batteries, single measures of global cognition, episodic memory and executive function.

A summary of the result of the primary meta-analysis along with forest plots can be found in Fig. 3. Four studies were included within the primary analysis as they assessed cognition using a composite score derived from neuropsychological test battery. The nutrient formulas that combined *n*-3 PUFA and B vitamins demonstrated a significant improvement in cognitive function *v*. the placebo with no significant heterogeneity between studies (G = 0·23, 95 % CI 0·09, 0·37, *P* = 0·002, I^2^ = 0 %).

Five studies were included in the analysis of single measures of global cognition, these measures included the mini mental state examination, (Alzheimer’s Disease Assessment Scale-cognitive subscale), Clinical Dementia Rating Sum of Boxes and full-scale IQ. Values for the level of variability were not provided for the study by Scheltens *et al.* ^(36)^, thus this study could not be included. In the study by Andreeva *et al.* ^(40)^, cognitive function was assessed at the conclusion of the supplementation period, and no baseline assessment was conducted. As such this analysis was comparing absolute values for cognition between groups at the end of the study so no conclusions can be drawn as to whether the supplementation slowed the rate of cognitive decline over time so this study was not included within the meta-analysis. The combined *n*-3 PUFA and B vitamin interventions demonstrated a significant improvement in single measures of global cognition *v*. the placebo, with their being significant heterogeneity between studies (G = 0·28, 95 % CI 0·09, 0·47, *P* = 0·004, I^2^ = 56 %). Five studies included domain-specific measurements of episodic memory. The combined *n*-3 PUFA and B vitamin interventions in these studies demonstrated a significant improvement in cognitive function *v*. the placebo with their being significant heterogeneity between studies (G = 0·32, 95 % CI 0·13, 0·51, *P* = 0·001, I^2^ = 62 %). Seven studies included domain-specific measurements of executive function, no significant effect was demonstrated in response to combined intervention with *n*-3 PUFA and B vitamins, with their being significant heterogeneity between studies (G = 0·29, 95 % CI − 0·13–0·71, *P* = 0·17, I^2^ = 63 %).

### Sub-group meta-analysis by baseline cognitive health status

**Fig. 4.**
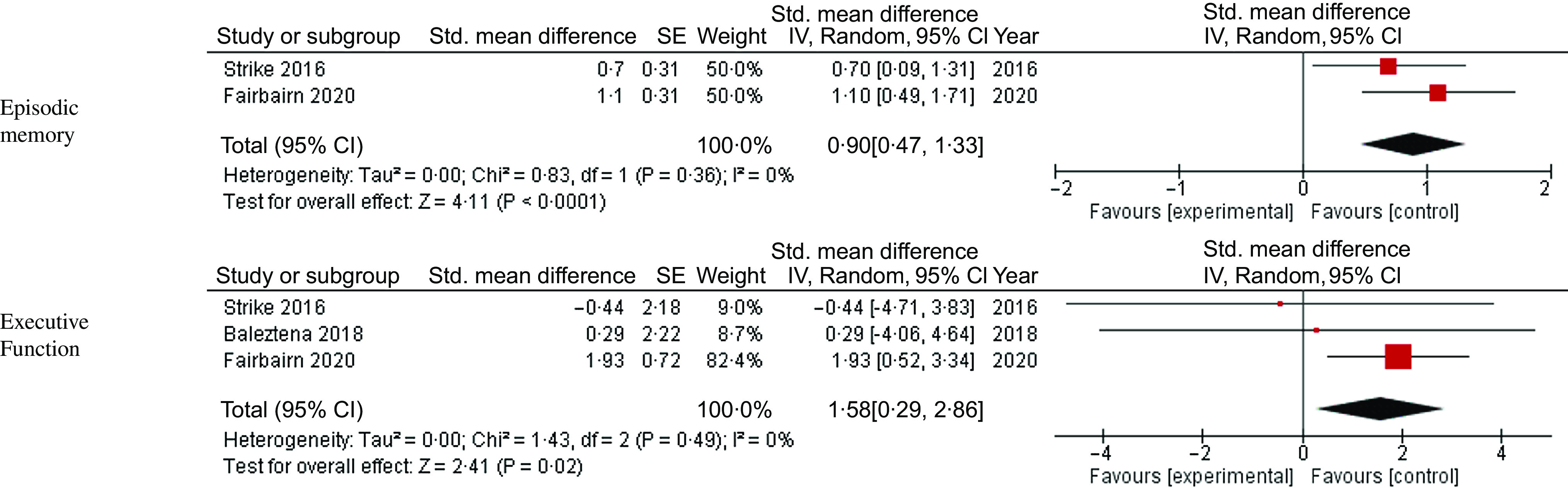
Meta-analysis and forest plots for the effects of multi-nutrient interventions containing both *n*-3 PUFA and B vitamins on episodic memory and executive function in cognitively healthy participants.

**Fig. 5.**
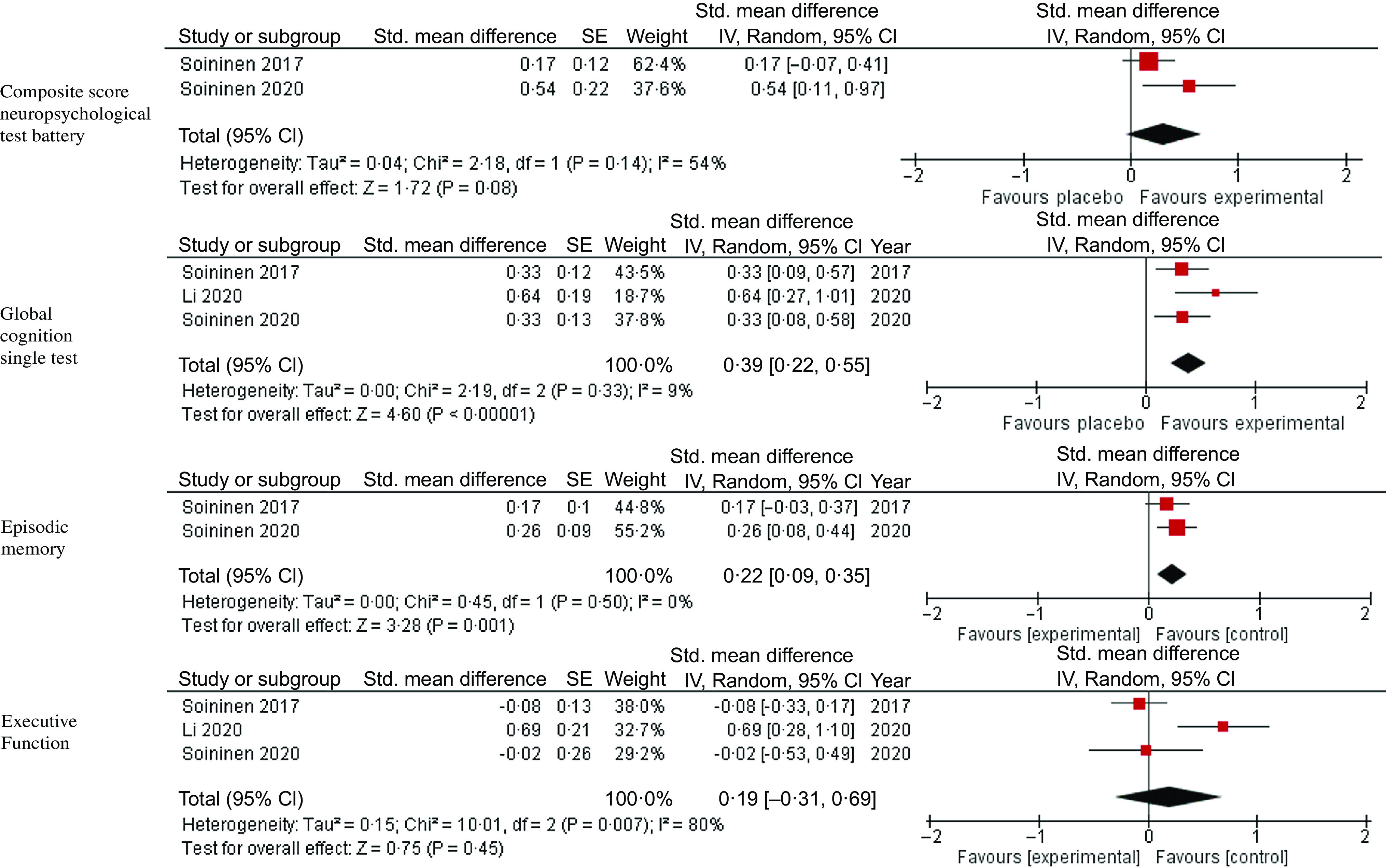
Meta-analysis and forest plots for the effects of multi-nutrient interventions containing both *n*-3 PUFA and B vitamins on composite scores from neuropsychological test batteries, single measures of global cognition, episodic memory and executive function in participants with mild cognitive impairment.

**Fig. 6.**
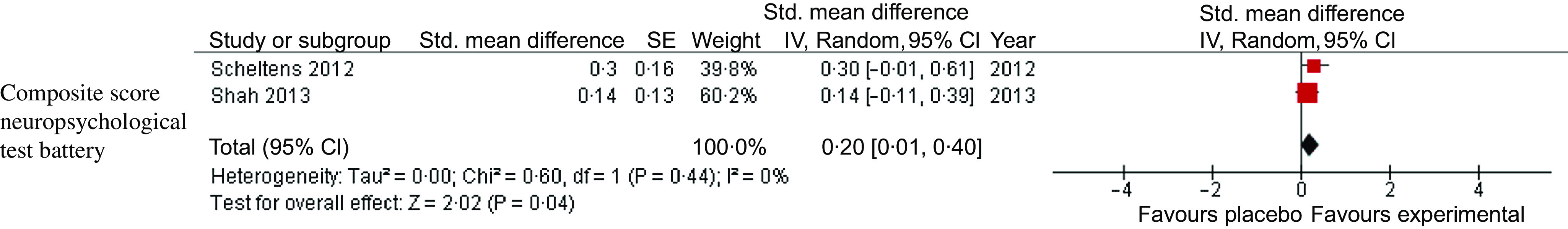
Meta-analysis and forest plots for the effects of multi-nutrient interventions containing both *n*-3 PUFA and B vitamins on composite scores from neuropsychological test batteries in participants with diagnosed Alzheimer’s disease.

A summary of the sub-group meta-analysis based on the cognitive health status of the included participants can be found in Fig. 4–6. Due to the small number of studies available some outcomes did not have more than one included study, as such only outcomes with at least two studies have been reported.

Three studies were included within the analysis for healthy participants^(31,33,38)^. The nutrient formulas that combined *n*-3 PUFA and B vitamins demonstrated a significant improvement in episodic memory (G = 0·90, 95 % CI 0·47, 1·33, *P* < 0·0001, I^2^ = 0 %) and executive function (G = 0·158, 95 % CI 0·29, 2·86, *P* = 0·02, I^2^ = 0 %) with no significant heterogeneity between studies. No studies in healthy adults reported a composite neuropsychological test battery scores, and only one study reported a single global cognition^(33)^.

Three studies were included within the analysis for mild cognitive impairment^(34,39,41)^. There was no significant effect of the nutrient intervention on the primary outcome of composite neuropsychological test battery score, with their being significant heterogeneity between studies (G = 0·31, 95 % CI -0·04, 0·66, *P* = 0·08, I^2^ = 54 %). The nutrient formulas that combined *n*-3 PUFA and B vitamins demonstrated a significant improvement in global cognition (G = 0·39, 95 % CI 0·22, 0·55, *P* < 0·00001, I^2^ = 9 %) and episodic memory (G = 0·22, 95 % CI 0·09, 0·35, *P* = 0·001, I^2^ = 0 %), with no significant heterogeneity between studies. There was no significant effect on executive function with their being significant heterogeneity between studies (G = 0·19, 95 % CI -0·31, 0·69, *P* = 0·45, I^2^ = 80 %).

Two studies were included within the analysis for participants with diagnosed Alzheimer’s disease^(35,37)^. The nutrient formulas that combined *n*-3 PUFA and B vitamins demonstrated a significant improvement in composite neuropsychological test battery score studies with no significant heterogeneity between studies (G = 0·20, 95 % CI 0·01, 0·40, *P* = 0·04, I^2^ = 0 %).

## Discussion

Results from this meta-analysis suggest a benefit of supplementing with nutrient formulas that contain both *n*-3 PUFA and B vitamins on global cognition and episodic memory with small to moderate effect sizes. The positive result from the primary analysis on global cognition assessed using composite scores derived from neuropsychological test battery were all from studies which had used the Fortasyn Connect formula. The Fortasyn Connect blend has shown promise in animal and *in vitro* models enhancing neuronal survival and hippocampal cholinergic neurotransmission by enhancing synaptic membrane formation^(45,46)^, as well as increases in neurogenesis and spatial memory compared with *n*-3 PUFA alone^(47,48)^. Despite this success at the pre-clinical stage, in the largest human trial to date, the Fortasyn Connect formula did not lead to significant improvements *v*. placebo in overall neuropsychological test battery score in older adults at the prodromal stage of Alzheimer’s disease^(34)^. A major limitation of this study was that the expected decline in cognitive function in the placebo group was 74 % lower than expected rendering the primary endpoint inadequately powered to detect any changes. This is an important consideration when evaluating the evidence for the role of interventions on cognitive decline as without adequate decline in the placebo group it is unlikely that any effect will be able to be detected. In the extension of this trial, where follow-up duration was 36 months, a more notable decline was observed within the placebo group, and a significant effect was observed for global cognition as well as episodic memory^(39)^. Overall, of the primary RCT included within this systematic review, not inclusive of the post hoc analyses, seven studies were classified as having a low risk of bias^(32,34–37,39,41)^, three had some concerns and one had a high risk of bias^(31,33,38)^. Of the studies that had some concerns a consistent area of bias was that cognitive tests commonly have multiple scales from which scores can be derived, it was not made clear in the methodologies or trial registries exactly how the scores would be calculated or whether any scales that were measured were omitted from the published work. Moving forward, it is important to include precise detail on the methods used for cognitive testing to help rule out any form of bias in the way results are reported. Although there were some concerns within this area. The study that had a high risk of bias was a result of having a notable number of missing data for the cognitive testing^(40)^. Seventy-two participants were unable to complete the cognitive testing, which was the equivalent of 4 % of the total sample size, it is unclear as to the precise reason for this but if this was due to cognitive decline there is a possibility this may have biased the results towards the null hypothesis given that a larger proportion of these participants were from the placebo group.

Results of the sub-group meta-analysis, which stratified studies by the baseline cognitive health status of the participants, found that *n*-3 PUFA and B vitamin multi-nutrient supplementation had a positive effect on executive function on those who were cognitively healthy at baseline, a result was that was previously not shown in the primary meta-analysis. There is currently no clear consensus in the literature as to whether *n*-3 PUFA improve executive function in older adults. For example, 900 mg DHA for 24 weeks had no effect on executive function in healthy older adults^(49)^, whereas 1320 mg EPA and 880 mg DHA for 26 weeks in sixty-five healthy older adults produced significant improvements^(13)^. Similarly, B vitamins have shown little promise in improving performance in this domain^(50)^. Although this result is promising only three studies were included within this meta-analysis, all of which had small sample sizes, and much of the weight was derived from one study^(38)^. More research providing both *n*-3 PUFA and B vitamins in healthy older adults could help to provide more definitive information as to whether supplementation could improve or preserve executive function. Results of the sub-group meta-analysis found that the Fortasyn blend had a positive effect on composite neuropsychological test battery scores in those with diagnosed Alzheimer’s but not in those with mild cognitive impairment. This result is somewhat surprising as previous evidence has indicated that *n*-3 PUFA and B vitamins alone have shown more promise in those with milder cognitive impairment^(14,27)^. There was a limited amount of available data for the analysis of mild cognitive impairment and much of the weight was derived from the aforementioned LipiDiDiet trial on the Fortasyn blend where the primary end point was rendered underpowered^(34)^. As such we suggest the results of the present sub-group meta-analysis should be considered preliminary at this stage; however, cognitive status at baseline should be an important consideration in future research that aims to assess the efficacy of *n*-3 PUFA and B vitamins on cognitive function in older adults. This recommendation to stratify based on baseline cognitive status would be aided by future research adopting a more standardised approach to assessing baseline cognition. This would lead to a more accurate and uniform classification of cognitive health status making future comparisons more robust.

Results from the post hoc analyses of previous studies are in support of the interaction between *n*-3 PUFA and B vitamins. Data from the post hoc analyses from the VITACOG study indicated that response to intervention with B vitamins is impacted by *n*-3 PUFA status with a combination of higher levels of *n*-3 PUFA alongside B vitamin supplementation conferring the greatest benefit to cognition^(21)^. Similarly, data from the OmegaAD demonstrated that response to *n*-3 PUFA supplementation appears to be diminished in those with elevated homocysteine^(42)^. There is, however, some discrepancy amongst the data from post hoc analyses as results from the FACIT trial suggested that B vitamin supplementation was more effective at lower levels of *n*-3 PUFA^(43)^. There are some important differences between the methods to note within this analysis. First, in the previously mentioned post hoc analyses, the participants already had some level of diagnosed cognitive impairment either having MCI or Alzheimer’s Disease, whereas the FACIT trial was conducted in healthy older women between the ages of 50–70 years. Within healthy participants, it can be common to observe lower levels of cognitive decline, which will impact the ability to detect changes. Indeed, within the FACIT trial, participants did not show any evidence of cognitive decline for global cognition, episodic memory or executive function in the placebo group, with declines in performance only being observed for processing speed. This make it difficult to draw direct comparisons to the VITACOG study where some decline was observed in the placebo group likely due to the increased susceptibility to decline of the participants recruited for the study, given that they were already displaying symptoms of cognitive impairment^(15)^. Another important distinction is that the effectiveness of the FACIT intervention, which was folic acid only, appeared dependent on EPA (low and middle tertiles), but not DHA; whereas in the VITACOG study, the effects of the mixed B vitamin intervention comprising folic acid, B_6_ and B_12_, appeared predominantly dependent on DHA. The blood fraction upon which *n*-3 PUFA were quantified in the FACIT trial was in the plasma cholesterol esters, where results were expressed as a percentage of total fatty acids, whereas within the similarly analysed VITCOG study *n*-3 PUFA were measured in total plasma and presented as absolute values^(51)^. *n*-3 PUFA are differentially incorporated into different blood fractions with EPA being preferentially incorporated into cholesterol esters and DHA into plasma phospholipids and triglycerides^(52)^, thus measuring cholesterol esters is a less reliable method for quantifying total *n*-3 PUFA status. The different fractions, different B vitamin interventions and the way in which the results have been expressed consequently make it difficult to draw comparisons between these two studies, as *n*-3 PUFA levels have been divided into tertiles so it is unclear as to whether there tertiles are actually comparable as the respective circulating levels could be quite different. There is a need for a more standardised approach to measuring and reporting fatty acid status within human trials to allow for more direct comparisons between studies^(53)^; however, it is understandable that this may be difficult to achieve in such post hoc analysis where samples have previously been taken and stored. It is important to note that although these post hoc analyses do provide some very interesting insight into the potential interaction between these nutrients, these studies were exploratory in nature, which lead to them being classified as having some concerns of bias in the RoB. As such they were not adequately powered to draw definitive conclusions, thus results should be considered preliminary at this stage. Furthermore, it is important to note that these studies did not provide both *n*-3 PUFA and B vitamins in their supplement formulas thus the post hoc analysis was looking into nutrient status from background diet. These results do, however, provide rationale for studies measuring the effects of a single nutrient intervention of *n*-3 PUFA or B vitamins to consider that the status of the other nutrient could impact the responsiveness of participants to supplementation.

The mechanisms underpinning this potential interaction between *n*-3 PUFA and B vitamins are currently not well understood; however, homocysteine has been shown to impact phospholipid and DHA metabolism by inhibiting methylation reactions that convert phosphatidylethanolamine enriched with DHA to phosphatidylserine, which in turn would influence phosphatidylserine synthesis by phosphatidylserine synthase-1 and phosphatidylserine synthase-2^(54)^. Furthermore, lower intakes of DHA could result in there being reduced availability of phospholipids for methylation. In particular, reduced phosphatidylethanolamine availability increases the S-adenosylmethionine, S-adenosylhomocysteine ratio, leading to the hypermethylation of histones *in vitro*. This leads to downstream effects on gene expression that increases stress-related responses and decreasing protein translation and mitochondrial function^(55)^. Furthermore, *in vitro* DHA has been shown to increase gene expression of 5-methyltetrahydrofolate reductase, the enzyme required to convert 5,10-methylenetetrahydrofolate to 5-methyltetrahydrofolate, which is an important factor in the re-methylation of homocysteine^(56)^. Whilst this mechanism is in support of the result from the VITACOG and OmegAD trials, it does not explain why results from the FACIT indicated that lower levels of *n*-3 PUFA increased responsiveness to the B vitamin intervention within the FACIT trial. A further exploratory analysis from this data set revealed that serum homocysteine was significantly associated with cortical amyloid beta in subjects with low erythrocyte *n*-3 PUFA levels, but not in participants with high *n*-3 PUFA levels. This is perhaps suggestive that elevated homocysteine could impact the development of Alzheimer’s disease in those with low levels of *n*-3 PUFA but have less impact in those with higher levels of *n*-3 PUFA. As such those with lower levels of *n*-3 PUFA may react more favourably to supplementation that subsequently lowers homocysteine. Consequently, the relationship between these nutrients deserves further exploration. A longitudinal study designed to examine the temporal association between homocysteine, different *n*-3 PUFA and cognitive outcomes would help to shed further light on these complex interactions.

### Strengths and limitations

To the best of the author’s knowledge, this is the first time the evidence base on combining *n*-3 PUFA and B vitamins has been systematically reviewed. This provides an up to date overview on the current evidence base and highlights the need to consider the complex relationship between these key nutrients in relation to cognition within the older adult. The review has focused on randomised double-blinded placebo control trails meaning that causality of interventions can be better established.

Whilst the focus of this study was to evaluate the effects of a combination of *n*-3 PUFA and B vitamins, studies that provided formulas with additional ingredients were also included. As such, it is not possible to determine whether these additional ingredients may have modified the response to such formulas. That being said, when looking at these formulas, *n*-3 PUFA and B vitamins arguably do stand out as having the strongest evidence base for potential efficacy. There is evidence to suggest that response to *n*-3 PUFA or B vitamin supplementation may be modified by baseline cognitive status. Participants who are healthy or at the earlier stages of cognitive decline have been shown to be more likely to respond to nutrients intervention *v*. those with diagnosed Alzheimer’s Disease or dementia^(27,57)^. Due to the limited available studies for the meta-analysis, participants were not stratified by dosage of nutrients or for the specific fatty acid composition of *n*-3 PUFA formulas. DHA is highly enriched within the brain and appears to be the more important bioactive *n*-3 PUFA in regard to prevention of cognitive decline^(5,58)^. Moreover, there are some data to suggest that combined dosages of 1 g EPA + DHA including a minimum of 580 mg DHA per day are required to elicit positive effects on episodic memory in adults with memory complaints and without^(58)^. Within the meta-analyses of the present study, all nutrient formulas had a DHA dosage of greater than 580 mg per day, which may have contributed towards the observed positive effects on cognition *v*. previous reviews of single nutrient interventions, which have included studies which provided lower dosages^(59)^. In regard to B vitamins, there are limited dose–response data for nutrient formulas; however, baseline homocysteine levels do appear to modify response to supplementation with those who have higher levels responding more favourably to supplementation^(15)^. A definitive threshold by which homocysteine needs to be lowered to observe a positive effect on cognition is yet to be established, but it has been suggested that attaining levels ≤ 10·1 μmol/l could be effective^(14,15)^. As we seek to develop a greater understanding of the potential interaction between *n*-3 PUFA and B vitamins, we also may need to consider the specific fatty acid composition and dosage provided in any nutrient blends, as well as the baseline homocysteine status of the participants.

A notable limitation that must be acknowledged with regard to this systematic review was the process by which articles were screened and data were extracted. Although a consensus opinion was sought from a second member of the research team for study inclusion and a proportion of the extracted data was checked for accuracy, a more robust approach would have been to run these processes independently and in duplicate.

There was notable heterogeneity in the methods that were used to assess cognition within the included studies as well as the domains that were tested. The decision was made to focus on studies that had used composite scores derived from neuropsychological tests batteries due as this method may be more sensitive to detecting changes^(24)^, with secondary analyses on single tests of global cognition and domain-specific tests of episodic memory and executive function. This meant that not all cognitive data from the included articles were entered into the meta-analysis.

Within the research into cognitive decline, there is currently no standardised protocol for cognitive testing. As the field moves forward, it is important to have more uniform methods for assessing cognitive status. The use of neuropsychological test batteries that form a composite score for multiple tests would likely be a good choice to use as a primary outcome for future research, with domain-specific tests being used as secondary outcomes. This approach would allow for the assessment of global cognition as well as allowing labs to detect any potential domain-specific effects.

### Conclusion

Taking into consideration of the literature described above, there is now evidence to suggest that providing nutrient formulas that contain both *n*-3 PUFA and B vitamins could be efficacious for preserving cognition in the older adults. A possible positive interaction between B vitamins and *n*-3 PUFA is promising and has largely not been considered in previous human trials. This may be of particular importance when we consider that vitamin B_12_ inadequacies are common in older adults^(60)^, and *n*-3 PUFA status has been demonstrated to be sub-optimal across multiple population groups^(61)^. More experimental work providing a combination of nutrients including both *n*-3 PUFA and B vitamins, in healthy older adults or those showing early signs of cognitive decline, is clearly warranted to better explore how nutrition as a whole can impact the trajectory of cognition in older adults. In particular studies, using a two-by-two factorial design investigating the effects of *n*-3 PUFA and B vitamins alone and when combined together would help to further explore this novel and promising interaction.
